# CBirTox is a selective antigen-specific agonist of the Treg-IgA-microbiota homeostatic pathway

**DOI:** 10.1371/journal.pone.0181866

**Published:** 2017-07-27

**Authors:** Katie L. Alexander, Jannet Katz, Charles O. Elson

**Affiliations:** 1 Department of Medicine, University of Alabama at Birmingham, Birmingham, AL, United States of America; 2 Department of Pediatric Dentistry, University of Alabama at Birmingham, Birmingham, AL, United States of America; 3 Department of Microbiology, University of Alabama at Birmingham, Birmingham, AL, United States of America; McGill University Health Centre, CANADA

## Abstract

Cultivating an environment of mutualism between host cells and the microbiota is vital, and dysregulation of this relationship is associated with multiple immune disorders including metabolic and skin diseases, asthma, allergy, and Inflammatory Bowel Disease (IBD). One prominent mechanism for maintaining homeostasis is the protective regulatory T cell (Treg)- Immunoglobulin A (IgA) pathway toward microbiota antigens, in which Tregs maintain homeostasis and provide critical survival factors to IgA^+^ B cells. In order to amplify the Treg-IgA pathway, we have generated a fusion protein, CBirTox, comprised of a portion of the carboxy terminus of CBir1, a microbiota flagellin, genetically coupled to Cholera Toxin B subunit (CTB) via the A2 linker of CT. Both dendritic cells (DCs) and B cells pulsed with CBirTox selectively induced functional CD4^+^Foxp3^+^ Tregs *in vitro*, and CBirTox augmented CD4^+^Foxp3^+^ cell numbers *in vivo*. The induced Foxp3 expression was independent of retinoic acid (RA) signaling but was inhibited by neutralization of TGF-β. CBirTox treatment of B cells downregulated mammalian target of rapamycin (mTOR) signaling. Furthermore, CBirTox-pulsed DCs induced substantial production of IgA from naïve B cells. Collectively these data demonstrate that CBirTox represents a novel approach to bolstering the Treg-IgA pathway at the host-microbiota interface.

## Introduction

Altered microbiota compositions have been associated with various metabolic diseases including obesity and diabetes, and a gut-brain axis in which the microbiota influences multiple neural pathways has recently been identified [1–3]. Furthermore, dysregulation of the intestinal host-microbiota relationship leads to elevation of immune responses towards harmless, commensal bacterial species and the onset of inflammation in Crohn’s Disease (CD) [4,5]. Considering there are ten-fold more bacterial cells in the human host than eukaryotic cells, it seems logical that multiple mechanisms of tolerance must be in place [6]. Tregs act as a major mediator of intestinal tolerance by suppressing aberrant immune activation through regulation of CD4^+^ T effector cell responses and proliferation [7–11]. Additionally, Tregs are a major T helper cell for IgA responses in the gut [12]. Tregs provide critical survival factors to IgA^+^ B cells, as well as TGF-β for B cell Class Switch Recombination (CSR) in the intestine, in order to induce a non-inflammatory, protective Treg-IgA pathway [12]. IgA aids in limiting systemic activation to commensal species by compartmentalizing microbial responses to the mucosal immune system [13]. Secretory IgA (SIgA), the secreted form of IgA, blocks adherence of invading pathogenic bacterial species to the epithelium by forming complexes with them in the lumen, thereby limiting potential for inflammation [14,15]. Promoting these regulatory mechanisms, particularly the Treg-IgA pathway, is an attractive therapy for disorders associated with a dysregulated host-microbiota relationship. CTB, the binding portion of CT, conjugated to self-antigens, has been demonstrated to promote regulatory functions, without the associated toxicity of the CT holotoxin. In addition to lowering quantities of pro-inflammatory cytokines and enhancing regulatory cytokines such as TGF- β, these constructs have been demonstrated to induce systemic immune tolerance and to protect from multiple autoimmune disorders including Type 1 Diabetes (T1D), Experimental Autoimmune Encephalomyelitis (EAE), and uveitis, through the induction of both Foxp3^+^ and Foxp3^-^ Treg cells in murine models [16–20]. While CTB-Ag constructs are well documented to enhance tolerance, mechanisms of this induction are not clearly defined.

To further investigate the mechanisms of CTB-Ag constructs, and their ability to enhance tolerance and the protective Treg-IgA pathway to microbiota antigens, we have constructed a fusion protein, CBirTox, in which a portion of the carboxy terminus of CBir1 flagellin is genetically fused to the A2 linker of CT and expressed recombinantly with CTB as a pseudo toxin. Considering both CT and CTB have previously been shown to inhibit proliferation of lymphocytes through direct binding [21], we hypothesized that CBirTox may enhance Foxp3 expression in CBir1 TCR Tg CD4^+^ T cells *in vitro* and *in vivo* through modulation of APC function. Indeed, both DCs and B cells pulsed with CBirTox selectively induced significant Foxp3 expression in CBir1 TCR Tg CD4^+^ T cells *in vitro* and *in vivo*. DCs from multiple tissues, including the spleen, mesenteric lymph node (MLN), and lamina propria (LP), induced similar amounts of Foxp3 expression in CBir1 Tg CD4^+^ T cells, demonstrating that CBirTox works on a variety of DC subsets to induce tolerogenic potential. While CBir1 TCR Tg T cells cultured with CBirTox pulsed APCs did not require the addition of exogenous TGF-β for Foxp3 induction, a classical requirement of *in vitro*-generated Tregs [22,23], neutralization of TGF-β via an inhibitor abrogated Foxp3 expression, suggesting high sensitivity to endogenous TGF-β signaling. B cells treated with CBirTox *in vitro* exhibit decreased expression of phosphorylated p70S6 kinase, a protein directly phosphorylated by mTOR [24], compared to B cells activated with antigen. Furthermore, CBirTox treated DCs induce considerable IgA production *in vitro* from a subset of naïve B cells via a mechanism that is in part dependent on RA signaling. Collectively, these data suggest that CBirTox modulates mTOR signaling in APCs to induce CD4^+^Foxp3^+^ Tregs directed at microbiota antigen, thereby establishing it as a novel inducer of the Treg-IgA pathway and tolerance.

## Results

### The CTB-A2-CBir1 fusion protein, CBirTox, activates CBir1 Tg T cells *in vitro*

**Fig 1:**
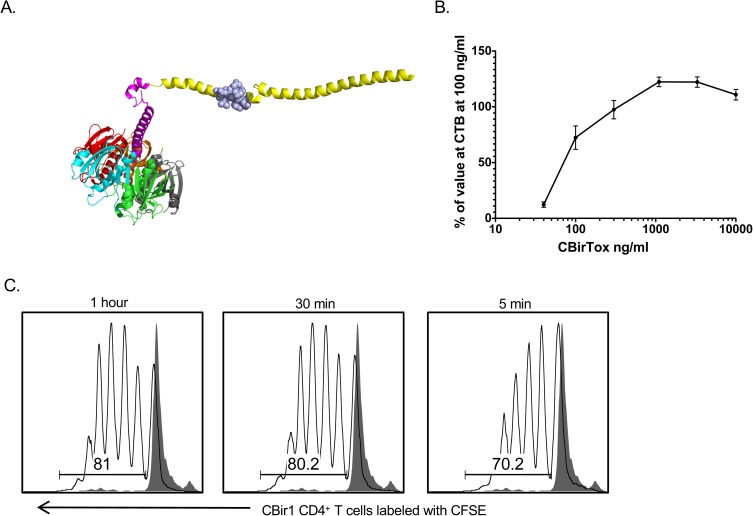
The CTB-A2-CBir1fusion protein, CBirTox, activates CBir1 TCR Tg T cells *in vitro*. (A) CBirTox is a genetic fusion protein consisting a portion of the C-terminus of CBir1 flagellin (yellow) that expresses the dominant CBir1 TCR transgenic epitope (blue), and is genetically fused to the A2 linker of CT and expressed recombinately with the CTB pentamer (multi-colored). (B) The pentameric structure of CTB in CBirTox as well as binding affinity were verified by GM-1 ELISA. Concentrations of the CBirTox pseudo-toxin were compared to the binding affinity of CTB at 100 ng/ml by GM-1 ELISA. Results are expressed as the mean ± SEM of 3 independent experiments. (C) CD11c^+^ DCs were pulsed with 1 μg/ml of CBirTox for 1 hour, 30 minutes or 5 minutes and then co-cultured 1:10 with CFSE labeled CD4^+^ CBir1 TCR Tg T cells for 3 days *in vitro* before flow cytometry analysis in order to verify biological activity of CBirTox. Representative flow of 3 independent experiments is shown.

The antigenic portion of the fusion protein CBirTox, which replaces the toxic A1 subunit of CT, is composed of a 261 base pair fragment of the carboxy terminus of CBir1 flagellin that expresses the dominant CBir1 TCR transgenic epitope, and is genetically fused to the A2 linker of CT and expressed recombinantly with CTB (Fig 1A). The CBir1 fragment was inserted into the pCTdelA1 vector, a CT vector lacking the toxic A1 subunit [25], using a process previously described [25]. The recombinant protein was purified with Ni-NTA agarose resin over a column, as the CTB pentamer binds robustly to immobilized nickel ions due to the His residues located at positions 13, 57, and 94 [26]. Because CTB possesses potent GM-1 ganglioside binding affinity, GM-1 ELISAs were used to delineate the binding affinity of CBirTox [27]. Indeed, CBirTox was determined to have similar binding affinity to GM-1 ganglioside as CTB (Fig 1B). CTB covalently linked to antigen has been reported to increase antigen presentation capacity and lower the threshold of antigen required for T cell activation [28]. In order to test the biological activity and antigen presentation properties of CBirTox, splenic CD11c^+^ DCs were pulsed with 1 μg/ml CBirTox, corresponding to 0.025 μg of CBir1 flagellin, for multiple time points and subsequently cultured with caroboxyfluorescein diacetate succinimidyl ester (CFSE) labeled CBir1 TCR Tg CD4^+^ T cells for three days *in vitro* before analysis with flow cytometry. DCs pulsed with CBirTox for as little as five minutes were able to induce significant proliferation in CBir1 TCR Tg CD4^+^ T cells, demonstrating that CBirTox efficiently presents antigen and is capable of activating antigen-specific CD4^+^ T cells *in vitro* (Fig 1C).

### DCs and B cells pulsed with CBirTox selectively induce CD4^+^Foxp3^+^ CBir1 Tg T cell *in vitro*

**Fig 2:**
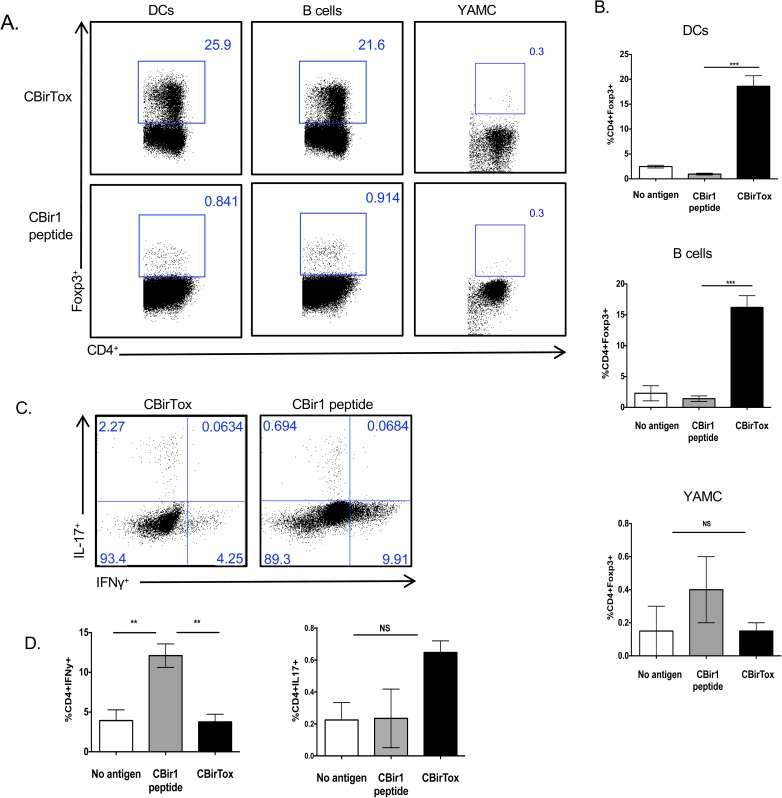
DCs and B cells pulsed with CBirTox specifically induce CD4^+^Foxp3^+^ CBir1 Tg T cell *in vitro*. (A-B) Splenic CD11c^+^ DCs were pulsed with 1 μg/ml CBirTox or CBir1 peptide for 1 hour while CD19^+^ B cells or YAMC cells were pulsed with 2 μg/ml CBirTox or CBir peptide for 2 or 4 hours, respectively, and co-cultured with CD4^+^CD25^-^ CBir1 Tg T cells for 4 days before flow cytometry analysis. Flow plots (A) are representative of 3–6 independent experiments; aggregate data shown (B). (C-D) CD19^+^ B cells were treated similarly to A and co-cultured with CD4^+^CD25^-^ CBir1 Tg T cells for 4 days before stimulation with PMA and ionomycin in the presence of Golgi Stop for 4 hours prior to staining for intracellular cytokines. Flow plots (C) are representative of 4 independent experiments. (D) Results are expressed as the mean ± SEM. *p<0.05, **p<0.005, ***p<0.0005, NS, not significant. Groups of three were analyzed using one-way ANOVA with Bonferroni’s post test, while groups of two were analyzed using Student’s unpaired t test.

Foxp3^+^ Tregs are important mediators of the immune response and function to control Teff cell responses as well as provide help to IgA^+^ B cells in the intestine [7,12]. In order to determine if CBirTox was capable of inducing Foxp3 expression in naïve T cells, CBirTox pulsed APCs were incubated with CD4^+^CD25^-^ CBir1 Tg T cells *in vitro*. Both splenic CD11c^+^ DCs and CD19^+^ B cells pulsed with CBirTox induced Foxp3 expression in approximately 20% of CD4^+^CD25^-^ CBir1 Tg T cells compared to less than 1% Foxp3 expression in cultures containing APCs pulsed with CBir1 peptide (Fig 2). Additionally, CBirTox pulsed CD19^+^ B cells induced substantial Foxp3 expression in naïve CD4^+^Foxp3^gfp-^ T cells sorted from B6.10BiT.Foxp3^gfp^.CBir1Tg mice, ensuring the expression of Foxp3 is not simply an expansion of contaminating Foxp3^+^ cells after isolation (S5 Fig). Significant Foxp3 induction is specific to treatment with the full CBirTox molecule, as APCs pulsed with CTB plus CBir1 peptide in concert did not generate substantial Foxp3 expression in CBir1 Tg T cells (S3 Fig). Young Adult Mouse Colon (YAMC) cells, which express MHC II strongly (S1 Fig), were treated with CBirTox in order to determine if an epithelial cell line could present the construct to T cells [29]. Interestingly, CBirTox pulsed YAMC cells did not induce Foxp3 expression in CBir1 CD4^+^ T cells (Fig 2A and 2B). CBirTox selectively induced Foxp3 expression, as both B cells and DCs pulsed with CBirTox did not promote production of IFN-γ after stimulation with PMA and ionomycin, in contrast to APCs pulsed with CBir1 peptide (Fig 2C and 2D). Additionally, APCs pulsed with CBirTox also did not induce IL-17 production in CBir1 Tg T cells after co-culture (Fig 2C and 2D). These data indicate CBirTox pulsed APCs selectively induce CD4^+^Foxp3^+^ Tregs *in vitro*.

### CBirTox induced Tregs share features with intestinal Tregs

Tregs are a heterogeneous population of cells expressing a variety of markers depending on their method of induction [30,31]. Tregs generated *in vitro* with the addition of TGF-β and IL-2 share similarities with Tregs directly isolated from the LP or adipose tissue, but they also exhibit extensive variations in their extended genetic profile [31]. In order to determine the phenotype of Tregs induced after CBirTox treatment, RNA was collected from sorted CD4^+^Foxp3^gfp+^ Tregs generated via co-culture of LPS-free CBirTox pulsed splenic CD19^+^ B cells and CD4^+^CD25^-^ CBir1 Tg T cells using B6.10BiTFoxp3^gfp^CBir1Tg mice (Table 1). CBirTox-generated Tregs express commonly associated Treg transcripts in addition to transcripts specific to Tregs generated *in vitro* with TGF-β, such as increased transcripts for EOS and decreases in the transcription factors JUN and FOS (Table 1) [31]. Interestingly, CBirTox-generated Tregs displayed upregulation of the suppressive molecule cytotoxic T lymphocyte-associated protein 4 (CTLA-4) and the chemokine receptor 4 (CCR4), two molecules that are typically expressed in LP Tregs [31]. Functionally, CBirTox-generated Tregs decreased IFN-γ and IL-2 production in subsequent cultures of freshly isolated CBir1 Tg CD4^+^CD25^-^ T effector cells, demonstrating suppressive function *in vitro* (S2 Fig).

**Table 1:** Genomic profile of CBirTox-generated Tregs.

Gene	Up (Fold)	Gene	Down (Fold)
**IL-2RA**	**294.4**	**IFNγ**	**457**
**GARP**	**200.9**	**IL-10**	**59.4**
**Foxp3**	**50.7**	**Eomes**	**21.5**
**SOCS2**	**25.7**	**Tbx21**	**17.1**
**EOS**	**20.5**	**LCN-2**	**16.5**
**CCR4**	**7.2**	**JUN**	**8.7**
**CTLA-4**	**5.9**	**FOS**	**4.8**

Splenic CD19^+^ B cells were pulsed with 2 μg/ml of LPS-free CBirTox for 4 hours, washed, and then cultured with splenic CD4^+^CD25^-^ T cells from B6.10Bit.Foxp3^gfp^.CBir1 mice. CD4^+^Foxp3^gfp+^ Tregs were flow sorted after four days and RNA was extracted and compared to RNA from naïve CD4^+^CD25^-^ T cells from B6.10Bit.Foxp3^gfp^.CBir1 mice using next-generation sequencing. Tuxedo suite software was used to analyze the data. Shown are selected gene transcripts associated with Tregs that are up or downregulated in CBirTox Tregs.

### CBirTox induces antigen specific CD4^+^Foxp3^+^ Tregs *in vitro* and *in vivo*

**Fig 3:**
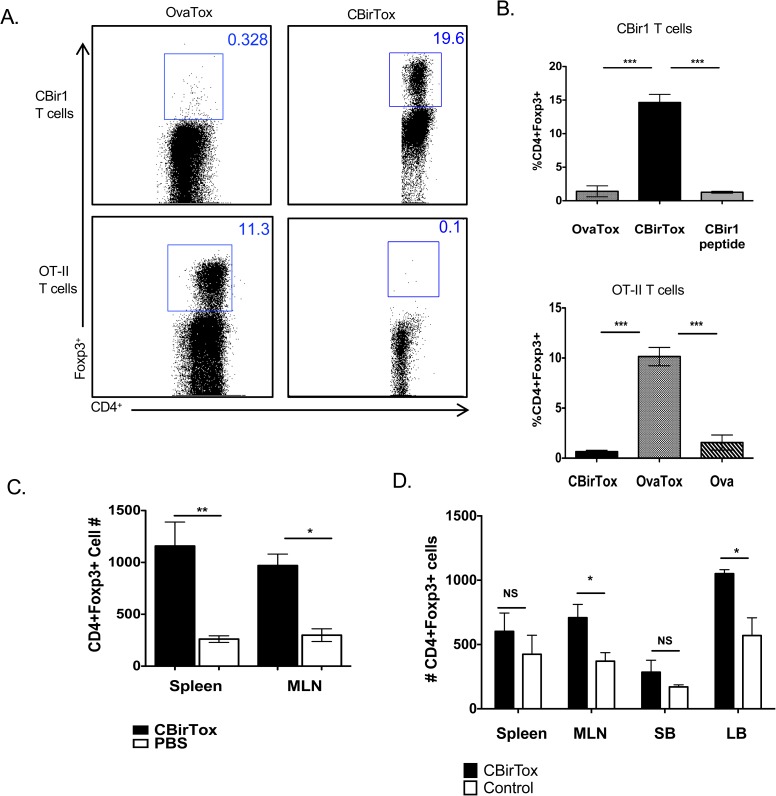
CBirTox induces antigen specific CD4^+^Foxp3^+^ Tregs *in vitro* and *in vivo*. (A-B) Splenic CD19^+^ B cells were pulsed with 1 μg of CBirTox or OvaTox, a pseudo-toxin similar to CBirTox, with a portion of OVA protein, containing OVAp323-339, replacing the CBir1 fragment, for 2 hours prior to co-culture with CD4^+^CD25^-^ CBir1 or OT-II Tg T cells for 4 days prior to flow cytometry analysis. Representative flow plots (A) are shown of 3 independent experiments (B). Results are expressed as the mean ± SEM. ***p<0.0005, Groups were analyzed using one-way ANOVA with Bonferroni’s post test. (C) CBirTox, 10μg, or PBS was injected i.v. into CBir1 TCR Tg mice. Spleens and MLNs were analyzed 7 days later for CD4^+^Foxp3^+^ T cells using flow cytometry analysis. Results represent 2 independent experiments. (D) CD4^+^CD25^-^ T cells were isolated from the spleen of B6.CBir1Tg/CD45.1 mice and injected (1x10^6^ cells) i.v. into B6.TCRβδ^-/-^ mice. Mice were then treated three times weekly i.p. with either 50 μg CBirTox or 5 μg CBir1 protein. Spleen, MLN, small bowel (SB), and large bowel (LB) were harvested and analyzed with flow cytometry. Results represent 3 independent experiments with 2–3 mice per group. Results are expressed as the mean ± SEM. *p<0.05. **p<0.005, NS, not significant. Groups of three were analyzed using one-way ANOVA with Tukey’s post test, and groups of two were analyzed using Student’s t test.

In order to determine the antigen-specificity of the CD4^+^Foxp3^+^ Treg induction, a second CTB-Ag construct, OvaTox, was developed. OvaTox was constructed in a manner similar to CBirTox, with a portion of OVA protein, containing the OT-II TCR epitope, fused 5’ to the A2 subunit in the pCTdelA1 vector. CD19^+^ B cells pulsed with either CBirTox or OvaTox were subsequently incubated with CD4^+^CD25^-^ CBir1 T cells or CD4^+^CD25^-^ OT-II T cells to determine antigen specificity. B cells treated with CBirTox induced CD4^+^Foxp3^+^ Tregs in CBir1 TCR Tg T cells only, while OvaTox pulsed B cells promoted CD4^+^Foxp3^+^ Tregs in OT-II T cells exclusively (Fig 3A and 3B). These data demonstrate the Treg generation by both CBirTox and OvaTox is antigen specific.

CTB-Ag constructs have been demonstrated to induce peripheral tolerance as well as protection from various autoimmune disorders through the induction of both Foxp3^+^ and Foxp3^-^ Treg cells *in vivo* [17,20]. In order to determine if CBirTox induced Foxp3 *in vivo*, CBir1 Tg mice were injected intravenously with CBirTox or PBS, and the CD4^+^Foxp3^+^ Treg population in the spleen and MLNs was analyzed one week later. CBirTox treated mice displayed augmented CD4^+^Foxp3^+^ Tregs in both the spleen and MLNs compared to control mice (Fig 3C). Importantly, CBirTox injection did not induce production of inflammatory cytokines (S4 Fig). Furthermore, intraperitoneal delivery of CBirTox augmented total CD4^+^Foxp3^+^ Tregs compared to delivery of antigen alone in B6.TCRβδ^-/-^ mice that had previously received CD4^+^CD25^-^ CBir1 TCR Tg T cells (Fig 3D). These data demonstrate that CBirTox functions to induce CD4^+^Foxp3^+^ Tregs *in vivo*.

### CBirTox induced expression of Foxp3 is dependent on TGF-β but independent of RA signaling

**Fig 4:**
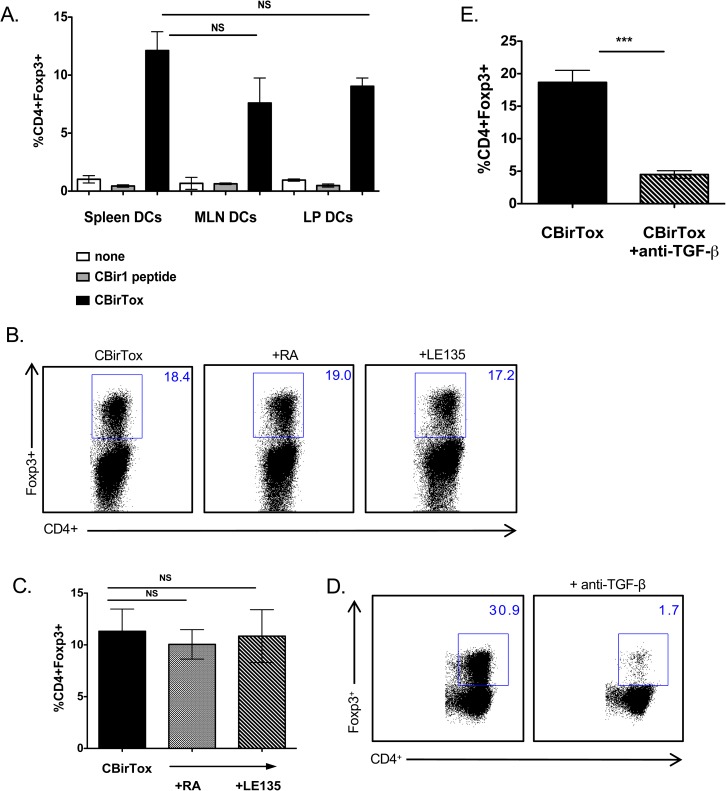
CBirTox induced expression of Foxp3 is dependent on TGF-β but independent of RA signaling. (A) CD11c^+^ DCs were isolated from the spleen, MLN or LP and pulsed with 1 μg/ml of CBirTox or CBir1 peptide for 2 hours prior to co-culture with CD4^+^ CBir1 Tg T cells for 4 days before flow cytometry analysis. Results represent 2–3 independent experiments (B-C) CD11c^+^ DCs were isolated from the spleen and pulsed with 1 μg/ml of CBirTox for 1 hour before co-culture with CD4^+^ CBir1 Tg T cells in the presence of 1 μM of the RA inhibitor LE135 or RA for 4 days. Representative flow plots (B) of 3 independent experiments (C) are shown. (D-E) Splenic CD19^+^ B cells were pulsed with 2 μg of CBirTox for 2 hours before co-culture with with CD4^+^ CBir1 Tg T cells with or without the presence of 10 μg/ml anti-TGF-β (1,2,3) for 4 days prior to flow cytometry analysis. Representative flow plots (D) of 4–5 independent experiments (E) are shown. Results of all experiments are expressed as the mean ± SEM. ***p<0.0005, NS, not significant. Groups of three or more were analyzed using one-way ANOVA with Bonferroni’s post test.

Tregs are differentiated in the presence of TGF-β and IL-2 in the periphery [9], though multiple other factors may aid in their induction, including retinoic acid (RA) and indolamine-2,3-dioxygenase (IDO) [32,33]. Specific DC subsets such as CD11c^+^CD103^+^CD11b^+^CX3CR1^-^ lamina propria DCs, which express retinaldehyde dehydrogenase 2 (RALDH2) and TGF-β, preferentially induce CD4^+^CD25^+^Foxp3^+^ Tregs [34]. Since splenic DCs, which do not typically express ALDH and thus generate RA [35], were proficient mediators of Foxp3 induction in CBir1 TCR Tg T cells after treatment with CBirTox, we hypothesized that DCs isolated from mucosal tissues, such as the lamina propria (LP), which have the ability to secrete RA, would induce superior amounts of CD4^+^Foxp3^+^ Tregs *in vitro*. Interestingly, DCs isolated from the spleen, mesenteric lymph node (MLN), and LP induced similar amounts of Foxp3 expression in CBir1 TCR Tg CD4^+^ T cells (Fig 4A). Considering the process for purifying DCs from the LP is more rigorous than isolating DCs from the spleen, entailing longer treatments with collagenase and more washes, it is possible that DCs preparations from this isolation incur more damage or contain an augmented amount of dead cells. To circumvent this issue and determine if RA plays a role in Foxp3 induction after CBirTox treatment of DCs, RA or the RA receptor inhibitor, LE135, was added to cultures of CBirTox pulsed DCs and CBir1 T cells. Neither RA nor LE135 altered the level of Foxp3 induction, indicating RA does not affect Treg generation in our system (Fig 4B and 4C). Interestingly, CBirTox pulsed DCs induce Foxp3 expression in the absence of exogenous TGF-β. To determine if TGF-β signaling plays a role in our system, anti-TGF-β was added to CBirTox pulsed APC-T cell cultures. Indeed, addition of anti-TGF-β abrogated Foxp3 expression, (Fig 4D and 4E) demonstrating a dependence on endogenous TGF-β signaling.

### CBirTox treatment decreases mTOR signaling in APCs

**Fig 5:**
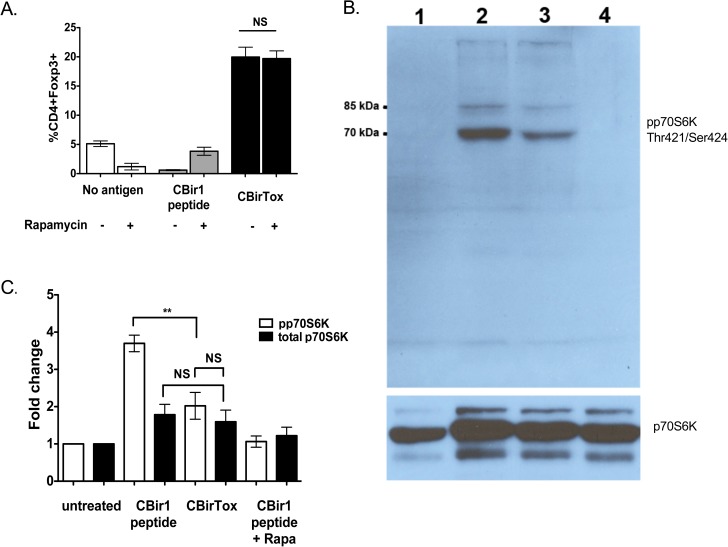
CBirTox treatment is associated with decreased mTOR signaling in APCs. (A) Splenic CD19^+^ B cells were pulsed with 1 μg/ml of CBirTox or CBir1 peptide for 1 hour before co-culture with CD4^+^ CBir1 Tg T cells with or without the addition of 30 nM rapamycin for 4 days before flow cytometry and other analysis. Results represent 3 independent experiments. (B) Splenic CD19+ were unpulsed (lane 1) or pulsed with 1 μg/ml CBir1 peptide (lanes 2,4) or with 2 μg/ml CBirTox (lane 3) for 4 hours before co-culture with CD4^+^ CBir1 Tg T cells for 48 hours. Rapamycin was added as a control (lane 4). B cells were then removed from culture, washed and lysates were prepared. Western blots were performed for phosphorylated p70S6 kinase (70,85 kDa) (Thr421/Ser424), top panel, and total p70S6 kinase, bottom panel. (C) Densitometry was performed on groups outlined in (B) for 4 independent experiments. Results are expressed as the mean ± SEM. **p<0.005, ***p<0.0005, NS, not significant. Groups were analyzed using one-way ANOVA with Bonferroni’s post test.

Previous work has demonstrated differential mTOR signaling in Teff and Treg populations, but little is known about how altered mTOR signaling in APCs may affect T cells. While mTOR signaling is necessary for induction of Th1, Th2, and Th17 subsets, mTOR deficient CD4^+^ T cells default to the Treg pathway [36]. Addition of anti-TGF-β inhibits the induction of Foxp3 in mTOR deficient T cells, suggesting sensitivity to endogenous levels of TGF-β, a characteristic similar to our experimental system [36]. In order to determine if mTOR may be inhibited in APCs after CBirTox treatment; rapamycin, the canonical mTOR inhibitor, was added to cultures of CBir1 TCR Tg CD4^+^ T cells with either CBirTox or CBir1 peptide pulsed B cells. Addition of rapamycin caused a slight, though not significant, increase in Foxp3 induction in CBir1 peptide cultures but elicited no change in CBirTox treated cultures (Fig 5A). Since rapamycin did not augment Foxp3 expression in CBirTox treated cultures, we inferred that CBirTox may downregulate mTOR signaling. To test this hypothesis, lysates were prepared from B cells treated with CBirTox or CBir1 peptide and compared to B cells treated with or without rapamycin. Western analysis was performed for the downstream target of mTOR signaling, p70S6 kinase. Indeed, CBirTox pulsed B cells displayed decreased phosphorylation of p70S6 kinase at Thr421/Ser424 compared to B cells pulsed with CBir1 peptide (Fig 5B). Significant downregulation of mTOR signaling by CBirTox was confirmed by densitometry (Fig 5C). Although CBirTox induced a substantial decrease in mTOR signaling, it did not cause total inhibition of phosphorylation of p70S6K, as was found in the control culture with rapamycin, the classical inhibitor of mTOR. Collectively, these data demonstrate that CBirTox treatment of B cells functions to limit mTOR signaling as a mechanism of promoting regulatory functions.

### CBirTox induces IgA production from naïve B cells *in vitro*

**Fig 6:**
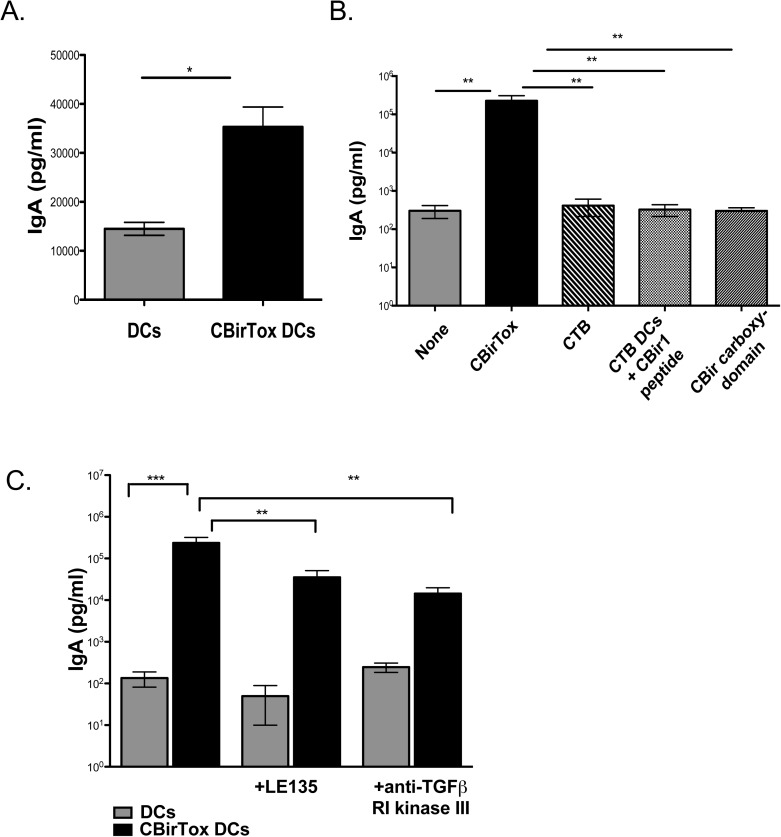
CBirTox induces IgA production from naïve B cells *in vitro*. Splenic CD11c^+^ DCs were pulsed with 1 μg/ml CBirTox or untreated for 4 hours before co-culture with CD19^+^ PP B cells (A). Supernatants were collected after 7 days and examined for IgA production via ELISA. Results are expressed as the mean ± SEM of 3–6 independent experiments. Groups were analyzed using student’s unpaired t test. (B) Splenic CD11c^+^ DCs were pulsed with 1 μg/ml CBirTox, 1 μg/ml CTB, 1 μg/ml CTB plus 1 μg/ml CBir1 peptide,1 μg/ml CBir1 carboxy-domain or untreated for 4 hours before co-culture with CD43^-^ splenic B cells at a 1:5 ratio. Supernatants were collected after 7 days and examined for IgA production via ELISA. Results are expressed as the mean ± SEM of 3 independent experiments. Groups were analyzed using one-way ANOVA with Tukey’s post test. (C) Splenic CD11c^+^ DCs were pulsed with 1 μg/ml CBirTox or untreated for 4 hours before co-culture with CD43^-^ splenic B cells at a 1:5 ratio with or without the addition of RA inhibitor LE135 or anti-TGF-β receptor I kinase III at 1 μM. Supernatants were collected after 7 days and examined for IgA production via ELISA. Results are expressed as the mean ± SEM of 4 independent experiments. Groups were analyzed using one-way ANOVA with Kruskal-Wallis correction. *p<0.05, **p<0.005, ***p<0.0005, NS, not significant.

CTB, as well as CTB-Ag complexes, have been previously shown to function as mucosal adjuvants and induce IgA responses *in vivo* [37,38]. In order to examine the regulation of IgA induction by CTB-Ag complexes, we developed an *in vitro* model system using the fusion protein CBirTox. Splenic DCs pulsed with CBirTox promoted IgA responses from CD19^+^ PP B cells after one week of co-culture, in the absence of any exogenous cytokine stimulation (Fig 6A). Furthermore, CBirTox-treated splenic DCs induced significant IgA production from naïve CD43^-^ splenic B cells, demonstrating that CBirTox is capable of *de novo* polyclonal induction of IgA in addition to expanding IgA^+^ B cell responses (Fig 6B). *In vitro*, CTB has been demonstrated to induce IgA production from naïve B cells via stimulation of MyD88 signaling through TLR agonists [39]. In order to determine if conjugation of CTB to CBir1 was necessary for initiation of IgA responses, DCs were pulsed with CTB, CTB plus CBir1 peptide or CBirTox. Splenic DC treated with CTB alone or mixed with CBir1 peptide did not induce significant amounts of IgA from splenic B cells, in contrast to splenic DCs pulsed with CBirTox, which induced substantial quantities of IgA in culture supernatants (Fig 6B). Collectively, these data indicate coupling of CTB to CBir1 is necessary for full activation of IgA responses in this *in vitro* system.

TGF-β is a critical factor in the terminal differentiation of B cells to IgA^+^ plasma cells [40]. In order to investigate the role of TGF-β in our *in vitro* system, the TGF-β signaling inhibitor, anti-TGF-β receptor I (RI) kinase III, was added to cultures of naïve B cells with CBirTox-pulsed or untreated DCs. Blockade of TGF-β signaling significantly decreased, but did not abolish, CBirTox-mediated IgA induction (Fig 6C). Additionally, LE135, an inhibitor of RA receptor signaling, was added to B cell cultures with CBirTox-pulsed or untreated DCs. Similarly, LE135 significantly downregulated, but not did nullify, IgA induction *in vitro* (Fig 6C). Altogether, these data indicate a role for TGF-β and RA in promotion of potentially protective CBirTox-mediated IgA responses, but also suggest additional mechanisms may also contribute to IgA induction *in vitro*. Taken together, the induction of polyclonal T cell independent IgA responses by the B cells in the presence of CBirTox may further enhance the homeostatic therapeutic potential of CBirTox by encouraging an anti-inflammatory mucosal environment.

## Discussion

Treatments of autoimmune disorders linked to dysregulated host-microbiota interactions commonly include the use of potent immune suppressants [41]. While these medications provide relief from excessive immune activation, they also have deleterious side effects, including increased risk of infection. A more attractive potential treatment of these disorders is the augmentation of regulatory pathways, including the protective Treg-IgA pathway [12]. The non-inflammatory Treg-IgA pathway supports a model in which Tregs cooperatively act with IgA^+^ B cells to maintain the microbiota, establishing mutualism. One way to encourage this regulatory pathway is through the induction of Tregs with CTB-Ag constructs [17,20]. CTB possesses high affinity binding to GM-1 ganglioside, which is located on nearly all nucleated cells, allowing it to efficiently increase the uptake, as well as presentation of antigen, by a wide variety of APCs [28,42–45]. Due to this unique binding capacity, CTB-Ag conjugates have been demonstrated to lower the threshold of antigen necessary to induce T cell activation *in vitro* by 10,000 fold; furthermore, they have been shown to induce tolerance induction at levels 100 fold less than treatment with free antigen alone *in vivo* [28,44]. While both CT and CTB have been demonstrated to have direct inhibitory effects on T cells, pretreatment of APCs with CT or CTB does not result in inhibition of T cell proliferation in subsequent cultures [21]. In this context, CTB-Ag constructs may function to increase Tregs by modulating APC functionality. In the current study, CBirTox treatment of B cells and DCs resulted in augmented Foxp3^+^ Tregs *in vitro* (Fig 2A and 2B). Importantly, CBirTox treatment did not promote the induction of Th1 or Th17 subsets (Fig 2C and 2D). The selective induction of CD4^+^Foxp3^+^ Tregs affords CBirTox the ability to specifically upregulate Tregs without inducing global T cell activation. This property, in conjunction with the fact that CBirTox-mediated induction of Tregs is directed against a microbiota constituent, makes CBirTox an attractive therapy during dysbiosis in the intestine, when inflammatory effector T cells outcompete tolerogenic Tregs.

CBirTox treatment of DCs from a variety of tissues including the spleen, MLN, and LP, conferred the ability to selectively induce Foxp3^+^ Tregs (Fig 4A). Surprisingly, splenic DCs were as effective as LP DCs in augmenting Tregs, despite the fact that LP DCs are considered more tolerogenic in nature [34,35]. RA does not play a role in this *in vitro* induction of Tregs, (Fig 4B and 4C) and therefore could partly explain this discrepancy, as the ability to make RA is a prominent feature of Treg-stimulating mucosal DCs [35,46]. Interestingly, CBirTox-Tregs have a transcriptional phenotype that shares similarities with both *in vitro* induced Tregs and LP Tregs (Table 1). The upregulation of the suppressive molecule, CTLA-4, which is commonly expressed on LP Tregs [31], is in line with the ability of CBirTox to exhibit suppressive functions *in vitro*. Indeed, CBirTox-generated Tregs decreased IFN-γ and IL-2 production in subsequent cultures of freshly isolated naive CBir1 TCR Tg CD4^+^CD25^-^ effector T cells (S2 Fig).

While addition of exogenous TGF-β was not necessary for CBirTox mediated induction of Foxp3 in CBir1 TCR Tg T cells, neutralization of TGF-β abrogated Treg generation (Fig 4D and 4E). This is in line with previous reports that demonstrate CD4^+^ T cells deficient in mTOR signaling have increased sensitivity to endogenous TGF-β and default to the Treg pathway, as they are unable to differentiate into Th1, Th2, and Th17 effector cell subsets [36]. The fundamental serine/threonine kinase, mTOR, is composed of two differentially regulated complexes, mTORC1, which is activated by PI3K signaling, and mTORC2, which is activated by growth factors [47]. mTOR plays a major role in regulating cell growth, proliferation, survival, and metabolism by sensing environmental signals [47,48]. Rapamycin, the classical inhibitor of mTORC1, is used clinically to downregulate T effector cell responses and promote Tregs in order to prevent graph rejection [49]. While mTOR has been extensively documented to affect T cell differentiation and function, its impact on APCs is less well defined [50]. DCs conditioned with rapamycin have been shown to have differing effects on immune responses. Inhibition of mTORC1 in DCs has been demonstrated to augment IL-12 production, the canonical Th1 cytokine, by decreasing IL-10 [49,51]. In contrast, rapamycin treated DCs have been shown to encourage induction of Foxp3^+^ Tregs and promote tolerance [52,53]. In B cells, complete inhibition of mTOR results in severe germinal center defects, including decreases in CSR and somatic hypermutation. Alternatively, low-level inhibition of mTORC1 and mTORC2 with mTOR kinase inhibitors (TOR-KIs) has been demonstrated to augment B cell proliferation and promote CSR [54]. The addition of rapamycin to CBirTox pulsed APC-T cell cultures did not augment Foxp3 induction (Fig 5A), suggesting that mTOR signaling may be altered in these cultures. Western analysis demonstrated that mTOR signaling is diminished in CBirTox-pulsed B cells (Fig 5B and 5C) compared to B cells treated with antigen alone. Herein, we propose that CBirTox downregulates mTOR signaling in B cells to encourage generation of Foxp3^+^ Tregs, but the exact molecular mechanism remains unknown.

The GM-1 binding affinity of CBirTox greatly increases its antigen presentation. For example, DCs pulsed for as little as five minutes with CBirTox were able to induce proliferation in CBir1 TCR Tg CD4^+^ T cells *in vitro* (Fig 1C). Furthermore, due to the ubiquitous expression of GM-1, B cells can present antigen irrespective of their B cell receptor affinity. Even with the enhanced antigen presentation provided by CBirTox, the generation of CD4^+^Foxp3^+^ Tregs *in vivo* (Fig 3C and 3D) was not as substantial as the *in vitro* results (Fig 2A and 2B). In this context, the GM-1 binding affinity of CBirTox may contribute to a loss of activity *in vivo*. It is possible that CBirTox may not have the opportunity to interact with DCs and B cells if it was absorbed by the epithelium upon entry. In agreement with this, YAMC cells were not able to activate CBir1 TCR Tg CD4^+^ T cells *in vitro* after CBirTox treatment; alternatively, YAMC cells pulsed with CBir1 peptide were able to activate CBir1 TCR Tg CD4^+^ T cells (S1 Fig). One possible mechanism for the increase in CD4^+^Foxp3^+^ Tregs in the colon after CBirTox treatment could be a decrease in pro-inflammatory cytokine signaling. CTB-Ag constructs have been demonstrated to modulate cytokine levels *in vivo*, including downregulation of IL-6 and IFN-γ, in various model systems [17,18]. IL-6 is an important modulator of the balance between Tregs and Th17 cells in the TGF-β rich environment of the intestine. TGF-β is necessary for the differentiation of both Treg and Th17 cells, but lower levels of IL-6 favor Treg over Th17 generation. Additionally, since IFN-γ is a positive regulator of Th1 differentiation and antagonist of TGF-β production, [55] CBirTox could contribute to a Treg-favoring, Teff-limiting cytokine milieu *in vivo*.

We have also demonstrated that CBirTox induces significant IgA production *in vitro* (Fig 6). This induction is independent of T cell help, but it is partly dependent on TGF-β and RA signaling, as neutralization of TGF-β with anti-TGF-β RI kinase III or RA with LE135 induced significant reductions in IgA production (Fig 6C). Previous work has suggested that CTB does not induce mucosal antibody adjuvant activity when uncoupled to antigen, in contrast to CT [25,56]. Alternatively, direct administration of CTB to the lungs of OVA-sensitized mice has been shown to promote IgA-mediated decreases in Th2 allergic characteristics [38]. In the current study, the induction of IgA via CBirTox was dependent on the coupling of CTB to the CBir1 antigen (Fig 6B). Splenic DCs treated with CTB mixed with CBir1 peptide did not induce IgA in subsequent cultures with B cells in the same manner as CBirTox pulsed DCs (Fig 2). This is in agreement with previous data suggesting coupling of CTB to a defined antigen is necessary for antibody promotion [25,56]. Collectively, these data demonstrate that CBirTox modulates mTOR signaling in APCs in order to induce Foxp3^+^ Tregs directed toward microbiota antigen, in addition to increasing IgA production from B cells *in vitro*. Therefore, CBirTox is a potent stimulator of the homeostatic Treg-IgA pathway and novel treatment option for multiple immune disorders associated with microbiota dysbiosis.

## Materials and methods

### Mice

C57BL/6 (B6) and B6.OT-II TCR Tg (OT-II) mice were purchased from Jackson laboratories. B6.CBir1 TCR Tg (CBir1-Tg) and B6.CBir1 TCR Tg/CD45.1 mice were generated at the University of Alabama at Birmingham (UAB) [12]. B6.10BiTFoxp3^gfp^. CBir1Tg mice were kindly provided by Dr. Craig Maynard, UAB [57]. All mice were maintained on a standard animal diet (Harlan) in the Animal Facility at UAB under specific pathogen free conditions in closed top cages in Thoren racks. Mice were monitored daily for in vivo experiments and weekly for husbandry purposes. Mice were anesthetized with isoflurane before in vivo treatment and the CO2-mediated method of sacrifice was employed using an ARP approved regulator. All experiments were reviewed and approved by the Institutional Animal Care and Use Committee of UAB.

### YAMC cell line

Young Adult Mouse Colon (YAMC) cells were grown at the tolerant temperature of 33°C in the presence of IFNγ (1 unit/ml, Peprotech) in complete RPMI 1640 with L-gluatamine (Cellgro) media supplemented with penicillin and streptomycin (Cellgro) in 5% fetal bovine serum (Atlanta Biologicals) (29). Before conducting assays, YAMC cells were washed with PBS and moved to the non-permissive temperature of 37°C in IFNγ-free media, and allowed to rest for 12–24 hours. All experiments were performed on cell cultures under 20 passages.

### CBirTox/OvaTox

CBir1 protein was cut using the restriction the enzymes NcoI and XhoI (NEB) at AA residues 407 and 493. The 261 base pair fragment containing the CBir1 Tg TCR epitope (residues 456–475) was ligated into the CTB vector pCTDA1 (described previously JI 4322–4332 1995). Briefly, the construct was grown in BL21(DE3) *E*. *coli* (Novagen) and induced with IPTG. Cells were shocked with MgSO_4_ and the supernatant was collected and incubated with Ni-NTA coupled to Sepharose CL-6B resin (Novagen) and purified over a column. The recombinant protein was eluted with imidazole and concentration using Amicon Ultra 15 Centrifugal Units (Millipore). OVA protein was cut using the restriction enzymes NcoI and XhoI at residues 301 and 385, which contains the OT-II TCR epitope, (AA residues 329–337) and ligated into the vector pCTDA1 before purification in a manner similar to CBirTox.

### GM-1 ELISAs

96-well plates were coated with 100 ng/ml GM-1 in PBS and incubated overnight at 4°C. After washing with 0.05%Tween 20 in PBS, the plates was blocked for 1 hour at RT with 1% bovine serum albumin (BSA) in PBS. Preparations of CBirTox and OvaTox were added to the plates in serial dilutions with CTB serving as the standard for 2 hours at RT. After washing, anti-CTB (derived from mice immunized with CTB) was added at a dilution of 1:250,000 for 1 hour before washing and treating with anti-mouse IgG conjugated to biotin (KPL) for 1 hour. Plates were then washed and strepavidin was added at 1:4000 for 30 minutes. Plates were developed with 3,3’, 5,5’ Tetramethylbenzidine (TMB) substrate (KPL) with the absorbance read at 450 nanometers.

### IgA studies

CD11c^+^ DCs were isolated from spleens of C57L/6 or B6.IgA KO mice and incubated with 1 μg/ml of CBirTox, CTB, or CTB plus CBir1 peptide for 3–4 hours at 37°C. After washing, the treated DCs or untreated DCs were co-cultured at a 1:5 ratio with CD43^-^ C57BL/6 naïve B cells for 7 days at 37°C in complete 1640 RPMI with L-gluatmaine media, supplemented with penicillin (1U/ml), streptomycin (100 μg/ml), 2.5 μM 2-ME and 10% fetal bovine serum (FBS) (Atlanta Biologicals) with or without the addition of 1 μM LE135 (Tocris) or anti-TGF-β R1 kinase inhibitor III (Calbiochem). Supernatants were collected and analyzed for IgA via ELISAs. Capture ELISAs were performed using anti-mouse IgA and anti-mouse biotin-labeled IgA (KPL).

### Isolation of lymphocytes and *in vitro* studies

CD4^+^CD25^-^ or CD4^+^ T cells were isolated after mechanical disruption of murine spleens by using the CD4^+^CD25^+^ regulatory T cell isolation kit (Miltenyi Biotec) or CD4^+^ BD IMag beads, respectively, via manufacturer’s instructions. B cells were isolated from murine spleens after mechanical disruption by enriching for CD19^+^ B cells labeling with CD19^+^ magnetic beads and processing with MACs columns (Miltenyi Biotec). To isolate DCs, spleens were treated with collagenase and processed using a spleen dissociation kit (Miltenyi Biotec) and subsequently labeled with CD11c^+^ magnetic beads (Miltenyi Biotec). DCs or B cells were pulsed with 1 or 2 μg/ml of CBirTox for 1–2 hours, respectively, at 37°C. After washing with PBS, DCs and B cells were cultured with CD4^+^CD25^-^ or CD4^+^ CBir1 Tg T cells at a ratio of 1:10 or 2:1, respectively with or without the addition of anti-TGF-β (1,2,3) (1D11) (R&D Systems), LE135, retinoic acid (Tocris), or TGF-β R1 kinase inhibitor III (Calbiochem) in complete 1640 RPMI with L-gluatmaine media, supplemented with penicillin (1U/ml), streptomycin (100 μg/ml), 2.5 μM 2-ME and 10% fetal bovine serum (FBS) (Atlanta Biologicals).

### Flow cytometry

Single cell suspensions were washed and stained for 30 minutes at 4°C with a combination of the following antibodies: CD44 (IM7)-PerCp/Cy5.5, CD69 (H1.2F3)-APC, CD4 (RM4-5)-PeCy7, PE, FITC, Pacific Blue, CD25 (PC61)-Pacific Blue, PerCP/Cy5.5 (Biolegend), washed and fixed in 1% paraformaldehyde or permeabilized with the Foxp3 staining buffer set (eBiosciences) and stained intracellularly with Foxp3 (FJK-16s) -PeCy7, APC for 30 minutes at 4°C. For cytokine staining, cells were first stimulated with phorbol 12-myristate 13-acetate (50 ng/ml, Sigma) and ionomycin (750 ng/ml, Sigma) in the presence of Golgi stop (10 μg/ml, BD Biosciences) for 4 hours before staining with IFNy (XMG1.2) -PE, APC or IL-17A (TC11-18H10.1)-PE, APC for 30 minutes at 4°C. Dead cells were excluded by staining with Live/Dead Aqua Amine dye (Invitrogen) during the extracellular staining step. Flow cytometry analysis was preformed on a BD LSR II and analyzed with FlowJo software (TreeStar).

### Suppression assay and cytokine ELISAs

CD4^+^CD25^-^gfp^-^ B6.10Bit.Foxp3gfp.CBir1Tg T cells were cultured with CBirTox-treated DCs for 5 days in order to induce Foxp3^+^ Tregs. CBirTox-generated Tregs were isolated by gating (FACsAria) on CD4^+^Foxp3-gfp^+^ cells. CBirTox-Tregs were cultured with freshly isolated CD4^+^CD25^-^ B6.CBir1TCR Tg T cells, B6 CD11c^+^ DCs and CBir1 peptide for 3 days. Supernatants were collected for IFNγ and IL-2 measurements via ELISAs. Capture ELISAs were performed using antibodies and standards purchased from BD Biolegend according to the manufacturer’s instructions.

### *In vivo* studies

CD4^+^CD25^-^ T cells were isolated from the spleen of B6.CBir1Tg/CD45.1 mice and injected (1x10^6^ cells) I.V. into B6.TCRβδ^-/-^ mice. Mice were then treated three times weekly i.p. with either 50 μg CBirTox or 5 μg CBir1 protein. Spleen, MLN, small bowel (SB), and large bowel (LB) were harvested and stained for live/dead markers, CD4, CD45.1, CD25, and Foxp3.

### RNASeq

CD4^+^CD25^-^ B6.10Bit.Foxp3gfp.CBir1Tg T cells were cultured with LPS-free CBirTox-treated B cells for 4 days to generate Foxp3^+^ Tregs. CBirTox Tregs were isolated by gating (FACsAria) on CD4^+^Foxp3-gfp^+^ cells. RNA was collected using the Ambion micro RNA extraction kit. Next-generation sequencing was performed using a HiSeq2000 at the Heflin Center for Genomic Science at UAB. Tuxedo suite software was used to analyze the data and compare gene transcripts from CBirTox-generated Tregs to naïve CD4^+^ T cells.

### Western analysis

CD19^+^ B cells were isolated from the spleens of B6 mice, treated with CBirTox and then washed, and subsequently cultured with B6.CBir1 Tg CD4^+^ T cells for 48 hours. CD4^+^ T cells were then removed from the culture using BD IMag CD4^+^ magnetic beads, and the B cells were washed 3 times before being lysed. Protein concentrations were determined using the Micro BCA Protein Assay Kit (Pierce). Equal amounts of protein were loaded onto a 10% Tris-HCl gel and separated by SDS-PAGE. Protein was then transferred to immobilon-P transfer membranes (Millipore) and detected with the following antibodies: p70S6K, pp70S6K (Thr421/Ser424) (Thr389), and HRP-linked rabbit or mouse anti-IgG (Cell Signaling). Densitometry was performed using an AlphaImager 2000 (Alpha Innotech).

### Statistics

Statistics were generated using Graphpad Prism software, version 5. Unpaired, two-tailed Student’s T tests were used to compare two groups, while ANOVA with Bonferroni or Tukey post-hoc tests were used to compare groups of three or more. P values > 0.05 were considered significant.

## Supporting information

S1 Fig YAMC cells express MHC II and activate CBir1 TCR Tg T cells *in vitro*. YAMC cells express MHC II and activate CBir1 TCR Tg T cells *in vitro*. In order to verify MHC II expression by epithelial cell line, YAMC cells were stained with isotype control or PE-MHC-II and analyzed via flow cytometry (A). Flow plots are representative of 3 independent experiments. YAMC cells or splenic CD11c^+^ DCs were pulsed with 1 μg/ml CBirTox for 4 hours, washed and then co-cultured with CD4^+^ CBir1 TCR Tg T cells and stained for the activation marker CD69 before flow cytometry analysis. Results represent 3 independent experiments and were analyzed using unpaired Student’s t test. **p<0.005.
(TIF)

S2 Fig CBirTox-generated Tregs have suppressive properties *in vitro*. CBirTox-generated Tregs have suppressive properties *in vitro*. Splenic CD11c^+^ DCs were pulsed with 1 μg/ml CBirTox for 2 hours before co-culture with CD4^+^CD25^-^ CBir1 Tg T cells isolated from the spleen of B6.10BitFoxp3.gfp.CBir1 TCR Tg mice. After 5 days, CD4^+^Foxp3gfp^+^ Tregs were isolated via flow cyotmetry and cultured with freshly isolated CD4^+^CD25^-^ CBir1 T cells labeled with CFSE (T conv) in the presence of 1 μg/ml CBir1 peptide and freshly isolated CD11c^+^ splenic DCs. After 3 days, supernatants were collected and examined for IFN-γ and IL-2 production via ELISA. Results represent 4 independent experiments and are expressed as the mean ± SEM. *p<0.05, NS, not significant. Groups were analyzed using one-way ANOVA with Bonferroni’s post test.
(TIF)

S3 Fig The CBirTox construct, not a CTB and CBir1 peptide mix, induce substantial Foxp3 expression in CBir1 TCR Tg T cells. CD4^+^ T cells were isolated from a CBir1 Tg mouse spleen and co-cultured with B6 CD19^+^ B cells pulsed with 1 ug/ml CBir1 peptide, CBirTox, CTB, CTB and CBir1 peptide together, or no antigen as a negative control for 3.5 days. Cultures were then stained with fluorescent antibodies and analyzed via flow cytometry. Representative flows plots of 2–3 independent experiments are shown.
(TIF)

S4 Fig CBirTox induces Foxp3^+^ Tregs *in vivo*. CBirTox, 10μg, or PBS was injected i.v. into CBir1 TCR Tg mice. Spleens and MLNs were harvested 7 days later and stained for CD4^+^Foxp3^+^ T cells (A) using flow cytometry analysis. Additionally, cells from the MLN were also stimulated with PMA and ionomycin and stained for IFNγ and IL-17 (B). Both (A) and (B) flow plots are representative of 2 independent experiments with groups of 2–3 each.
(TIF)

S5 Fig CBirTox induces de novo generation of Foxp3^+^ CBir1 Tg T cells *in vitro*. CD4^+^Foxp^gfp-^ T cells were flow sorted (A) or CD4^+^ T cells were isolated using BD Biosciences CD4^+^ isolation beads (B) from the spleen of a B6.10BiT.Foxp3^gfp^.CBir1 Tg mouse. The isolated T cells were cultured 1:2 with B6 splenic CD19^+^ B cells that had been pulsed with 2 μg/ml of CBirTox for 2 hours, B cells pulsed with 1 μg/ml of CBir1 peptide for 2 hours, or unpulsed B cells as a negative control for 3.5 days. Cells were then harvested and stained for flow cytometry. Resulting cells were then gated on live CD4^+^ T cells, and representative expression of CD25^+^ versus Foxp3^+^ expression is shown.
(TIF)
