# Supplementary figures and images for: CBirTox is a selective antigen-specific agonist of the Treg-IgA-microbiota homeostatic pathway
Source: PLoS One. 2017 Jul 27;12(7):e0181866. doi: 10.1371/journal.pone.0181866 (PMC5531474; doi:10.1371/journal.pone.0181866)

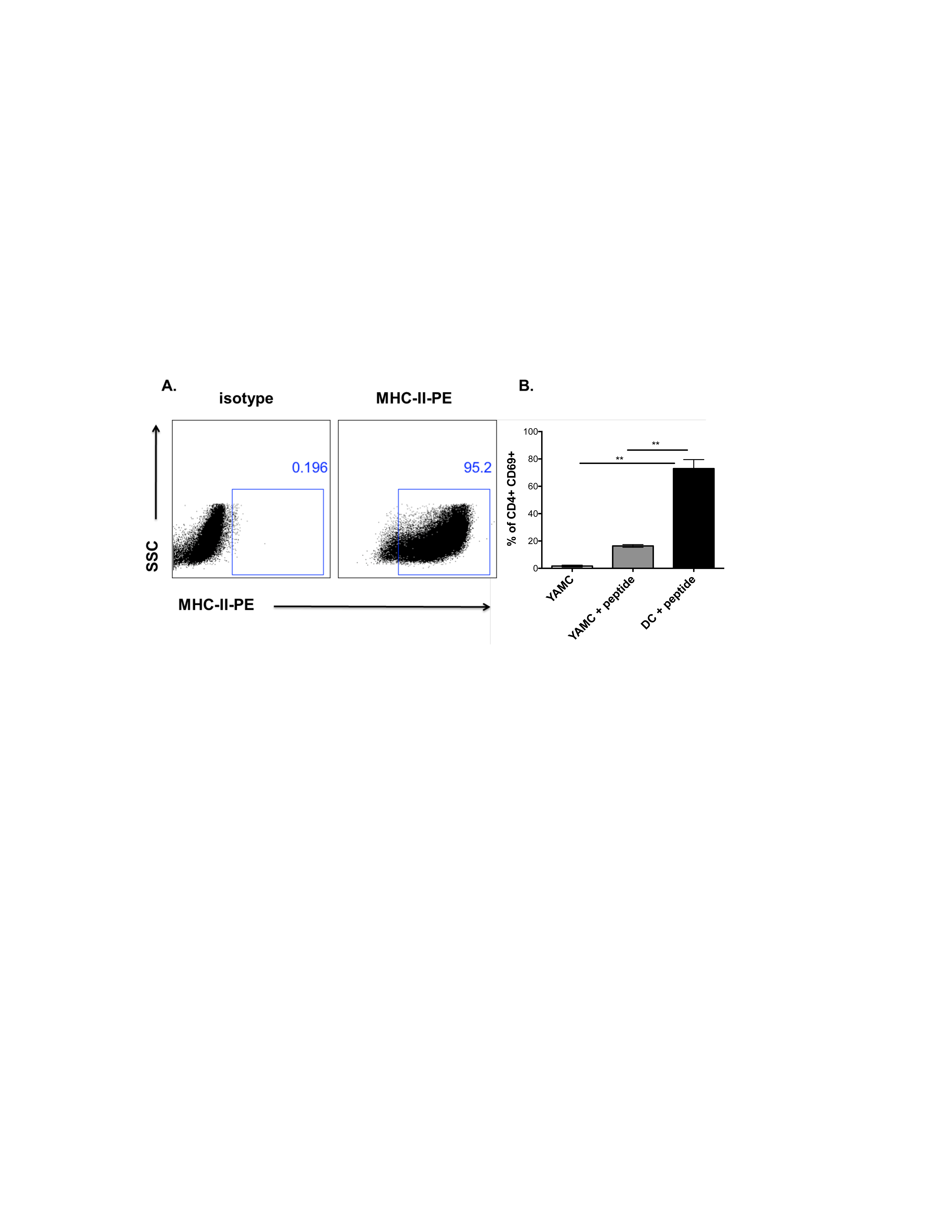

Supplement: S1 Fig — YAMC cells express MHC II and activate CBir1 TCR Tg T cells in vitro. In order to verify MHC II expression by epithelial cell line, YAMC cells were stained with isotype control or PE-MHC-II and analyzed via flow cytometry (A). Flow plots are representative of 3 independent experiments. YAMC cells or splenic CD11c+ DCs were pulsed with 1 μg/ml CBirTox for 4 hours, washed and then co-cultured with CD4+ CBir1 TCR Tg T cells and stained for the activation marker CD69 before flow cytometry analysis. Results represent 3 independent experiments and were analyzed using unpaired Student’s t test. **p<0.005. (TIF) [file pone.0181866.s001.tif]

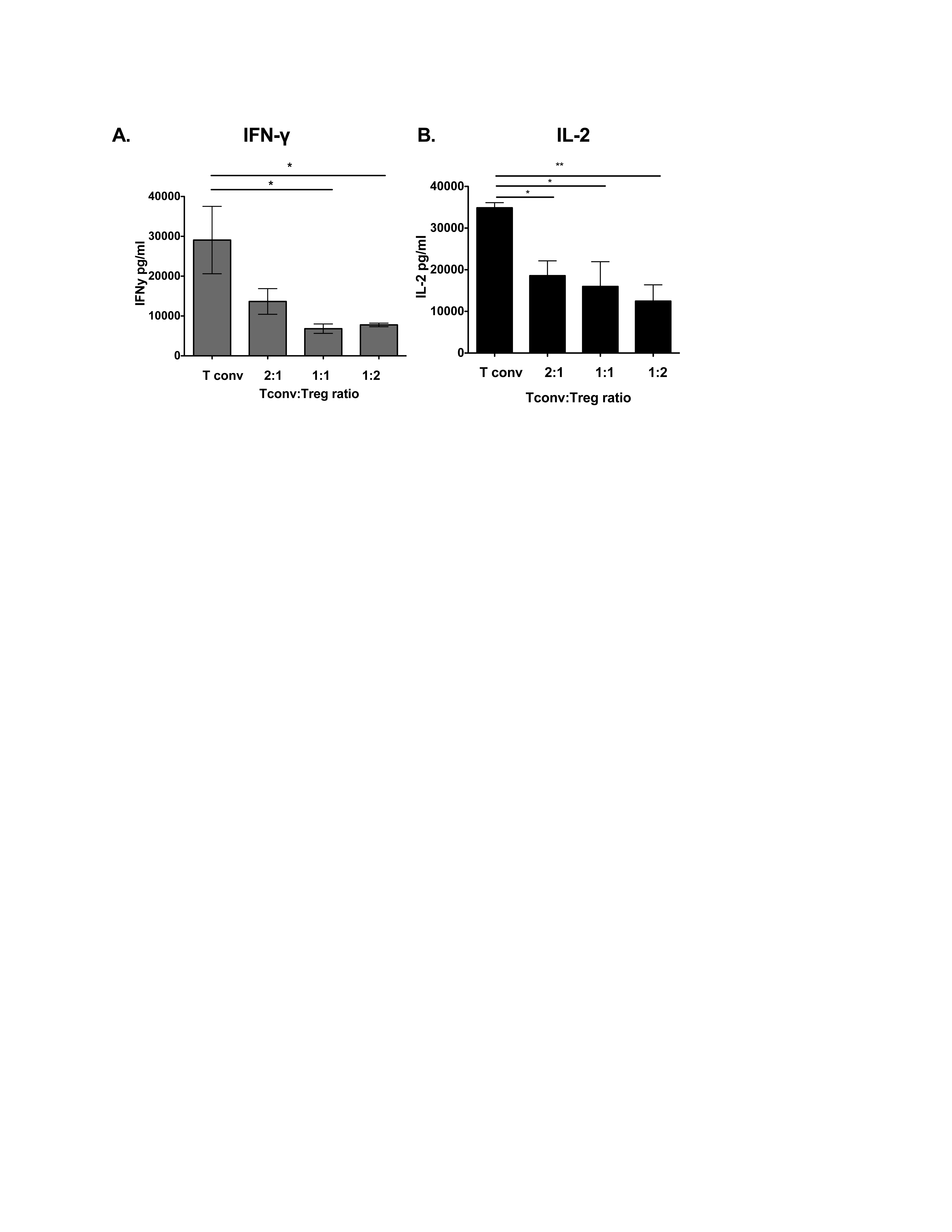

Supplement: S2 Fig — CBirTox-generated Tregs have suppressive properties in vitro. Splenic CD11c+ DCs were pulsed with 1 μg/ml CBirTox for 2 hours before co-culture with CD4+CD25- CBir1 Tg T cells isolated from the spleen of B6.10BitFoxp3.gfp.CBir1 TCR Tg mice. After 5 days, CD4+Foxp3gfp+ Tregs were isolated via flow cyotmetry and cultured with freshly isolated CD4+CD25- CBir1 T cells labeled with CFSE (T conv) in the presence of 1 μg/ml CBir1 peptide and freshly isolated CD11c+ splenic DCs. After 3 days, supernatants were collected and examined for IFN-γ and IL-2 production via ELISA. Results represent 4 independent experiments and are expressed as the mean ± SEM. *p<0.05, NS, not significant. Groups were analyzed using one-way ANOVA with Bonferroni’s post test. (TIF) [file pone.0181866.s002.tif]

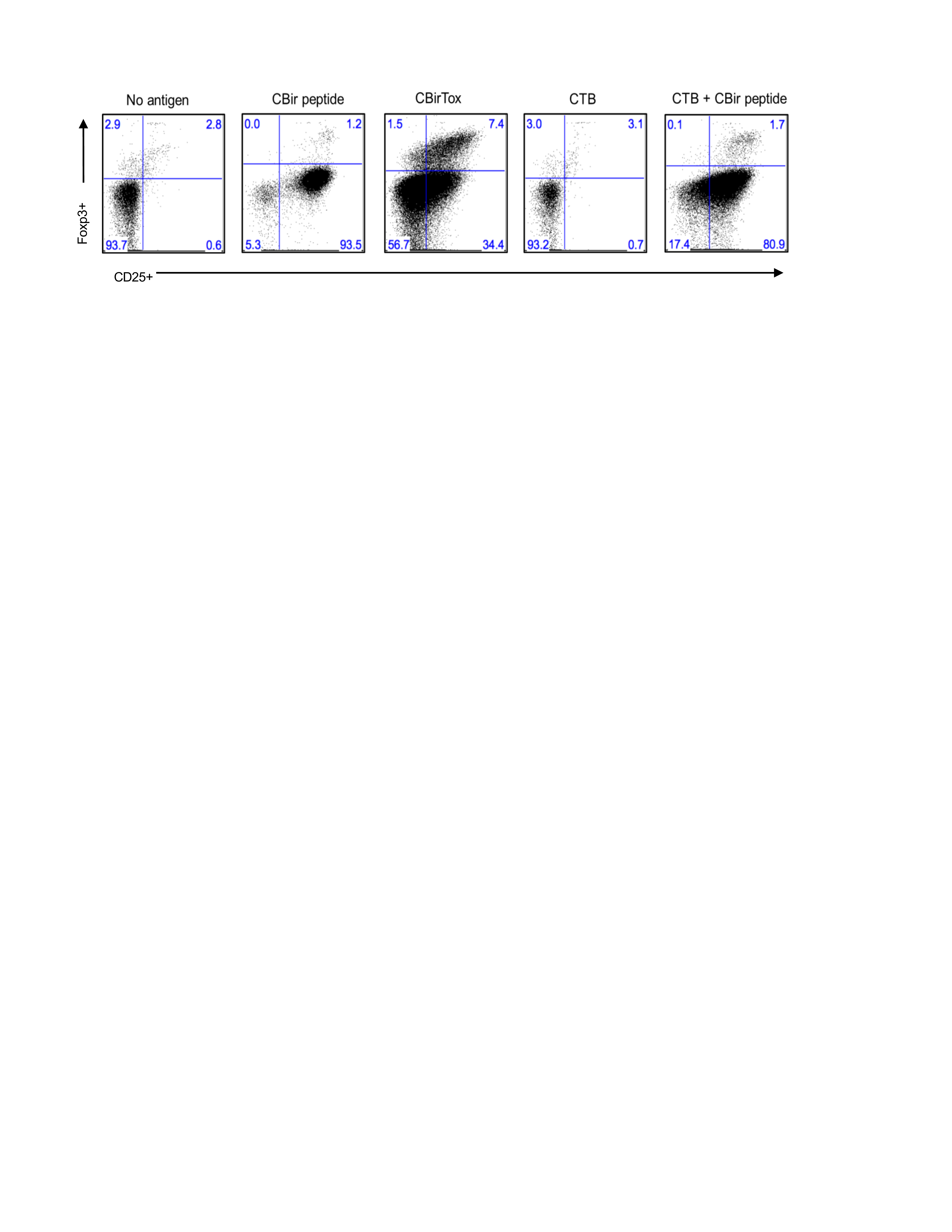

Supplement: S3 Fig — CD4+ T cells were isolated from a CBir1 Tg mouse spleen and co-cultured with B6 CD19+ B cells pulsed with 1 ug/ml CBir1 peptide, CBirTox, CTB, CTB and CBir1 peptide together, or no antigen as a negative control for 3.5 days. Cultures were then stained with fluorescent antibodies and analyzed via flow cytometry. Representative flows plots of 2–3 independent experiments are shown. (TIF) [file pone.0181866.s003.tif]

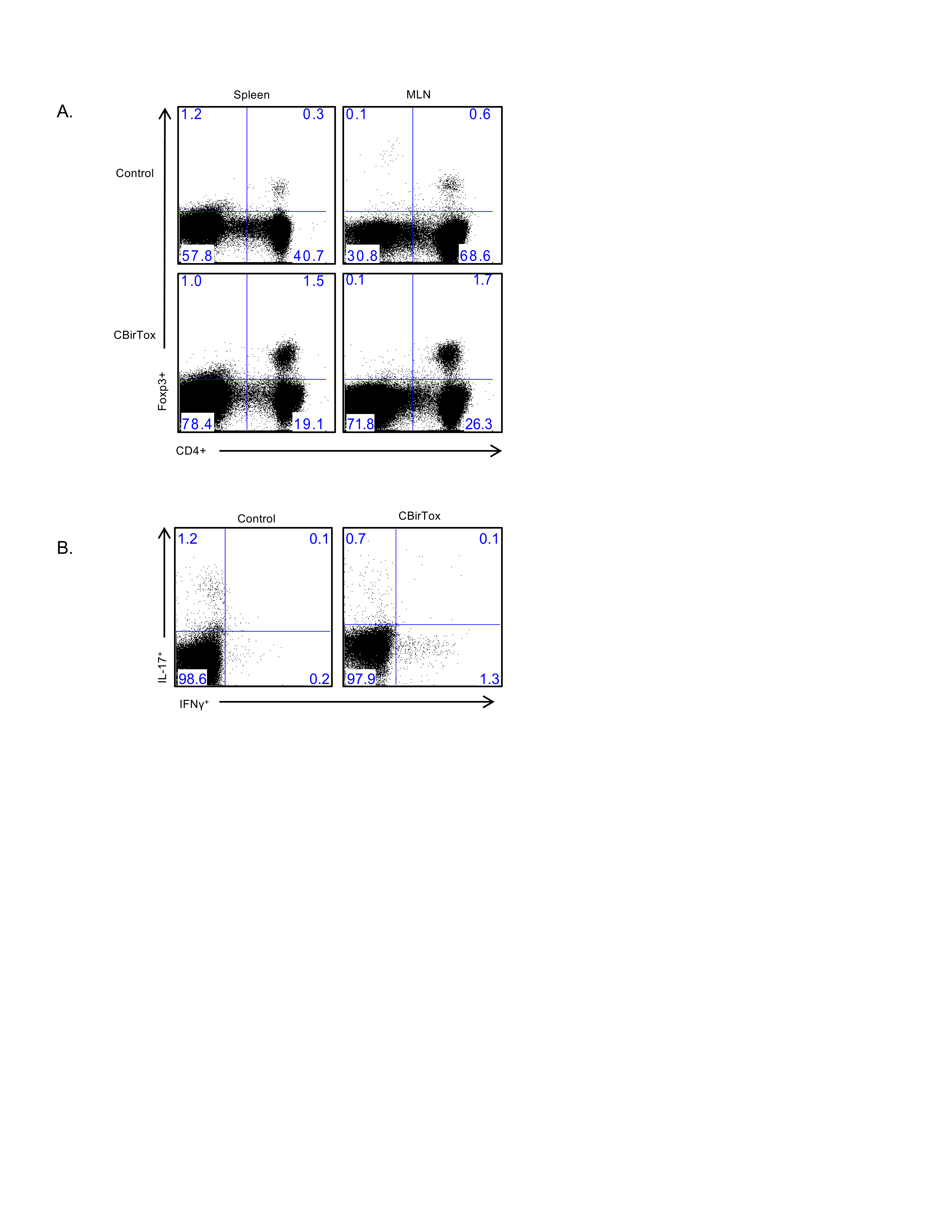

Supplement: S4 Fig — CBirTox, 10μg, or PBS was injected i.v. into CBir1 TCR Tg mice. Spleens and MLNs were harvested 7 days later and stained for CD4+Foxp3+ T cells (A) using flow cytometry analysis. Additionally, cells from the MLN were also stimulated with PMA and ionomycin and stained for IFNγ and IL-17 (B). Both (A) and (B) flow plots are representative of 2 independent experiments with groups of 2–3 each. (TIF) [file pone.0181866.s004.tif]

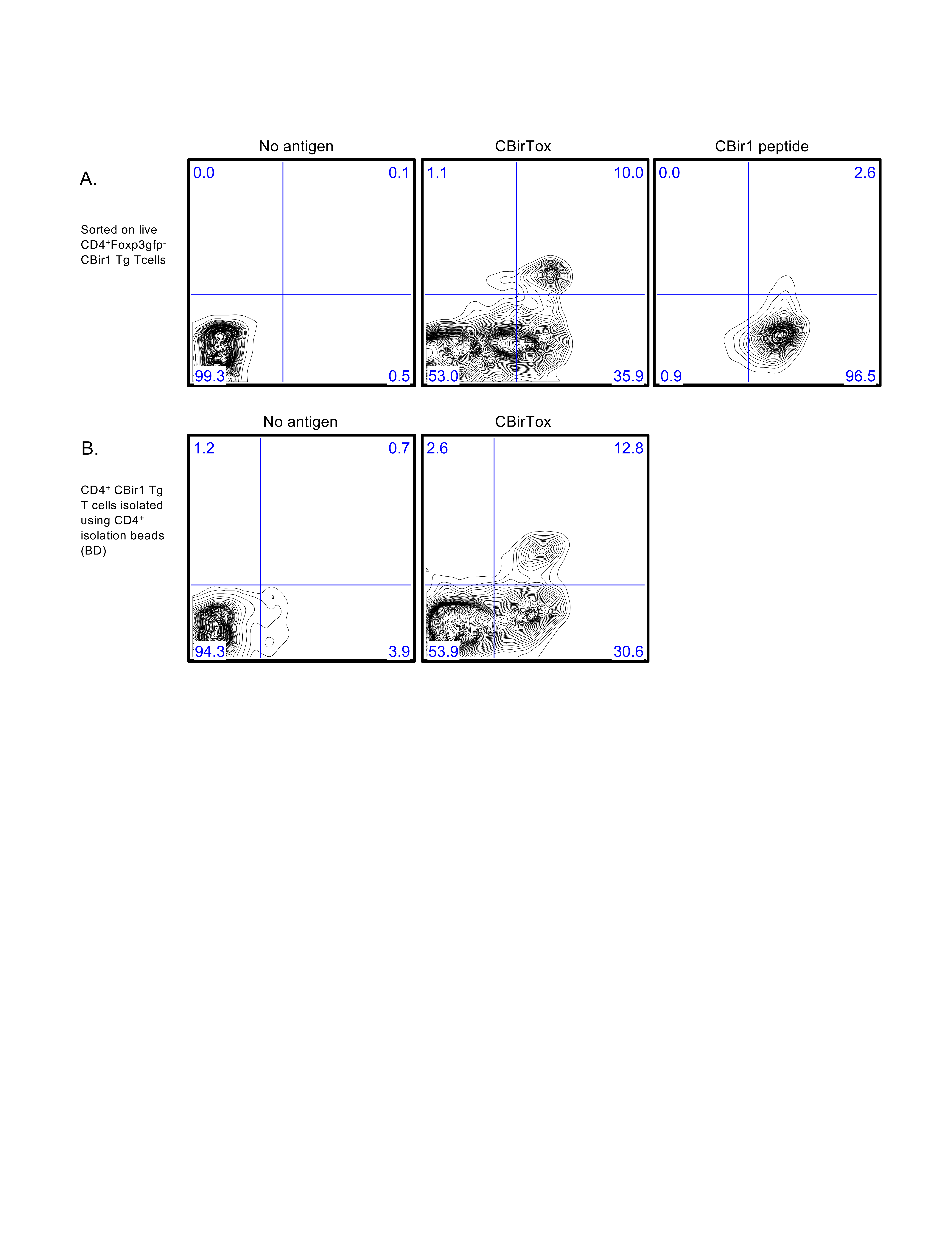

Supplement: S5 Fig — CD4+Foxpgfp- T cells were flow sorted (A) or CD4+ T cells were isolated using BD Biosciences CD4+ isolation beads (B) from the spleen of a B6.10BiT.Foxp3gfp.CBir1 Tg mouse. The isolated T cells were cultured 1:2 with B6 splenic CD19+ B cells that had been pulsed with 2 μg/ml of CBirTox for 2 hours, B cells pulsed with 1 μg/ml of CBir1 peptide for 2 hours, or unpulsed B cells as a negative control for 3.5 days. Cells were then harvested and stained for flow cytometry. Resulting cells were then gated on live CD4+ T cells, and representative expression of CD25+ versus Foxp3+ expression is shown. (TIF) [file pone.0181866.s005.tif]
